# Efficiency analysis of 67 Chinese research universities considering inter-university heterogeneity: Evidence from a meta-frontier network SBM DEA model

**DOI:** 10.1371/journal.pone.0331923

**Published:** 2025-09-18

**Authors:** Bo Cheng

**Affiliations:** 1 College of Physical Education and Health Management, Chongqing University of Education, Chongqing, China; 2 Graduate School, University of the East, Manila, Philippines; Southwestern University of Finance and Economics, CHINA

## Abstract

With the continuous increase in technology research and development investment, the overall operational efficiency of universities as the main body of scientific research has always been a focus of research. This study evaluates the efficiency of technology transfer in 67 Chinese universities directly affiliated with the Ministry of Education from 2016 to 2020. By integrating the meta-frontier analysis with the network Slacks-based Measure (SBM) Data Envelopment Analysis (DEA) approach, we assess the overall efficiency, stage-specific efficiency, and sources of inefficiency across different types of universities. Results indicate that while some institutions operate at the optimal frontier, the overall efficiency remains moderate, with the Technology Transfer and Application (TTA) stage consistently underperforming compared to the R&D stage. Significant heterogeneity exists among university types: normal, and medical & pharmaceutical universities demonstrate higher efficiency levels, whereas comprehensive, science and engineering, and agricultural and forestry universities exhibit notable inefficiencies, particularly in the TTA stage. Further decomposition reveals that technological gaps are the dominant source of inefficiency, especially in the later stage of the innovation process. Based on these findings, we propose targeted policy recommendations aimed at improving infrastructure, enhancing management practices, and tailoring reform strategies according to institutional type. This study contributes to the understanding of internal inefficiencies in university-led technology transfer and provides practical insights for policymakers and university administrators seeking to enhance the commercialization of academic research.

## 1. Introduction

In recent years, with the continuous advancement of the “Double First-Class” initiative in China’s higher education system, university research has become an increasingly important indicator of academic excellence and societal contribution [1]. According to the *2023 Statistical Bulletin on National Science and Technology Expenditure* jointly issued by the National Bureau of Statistics, the Ministry of Science and Technology, and the Ministry of Finance, China’s total expenditure on research and development (R&D) reached 3,335.71 billion yuan in 2023, reflecting a steady growth rate of 8.4% compared to the previous year. Among this, R&D funding allocated to higher education institutions amounted to 275.33 billion yuan—an increase of 14.1%, the highest among all executing entities—highlighting the growing importance of universities in the national innovation ecosystem. Notably, basic research funding reached 225.91 billion yuan in 2023, representing a year-on-year increase of 11.6%, and accounting for 6.77% of total R&D expenditure—the highest proportion on record [2]. Higher education institutions and government-affiliated research organizations remain the primary contributors to basic research, responsible for 60.2% and 31.6% of its growth, respectively. These figures underscore the significant progress made by Chinese universities in terms of R&D investment and their pivotal role in supporting national scientific and technological innovation.

However, alongside rapid development, substantial disparities have emerged across regions, institutional types, and structural dimensions. These imbalances pose new challenges to improving the efficiency and quality of university research performance. Currently, most evaluation systems focus primarily on quantitative indicators such as the number of publications, total research funding, patents granted, and awards received. While these metrics are easy to collect and implement, they suffer from several limitations. First, overreliance on quantity-based indicators may encourage a “publish-or-perish” culture that neglects the quality and real-world impact of research outcomes. Second, existing methods may not fully capture the relationship between inputs and outputs, which could result in rankings that overstate the performance of institutions with relatively high inputs but modest output efficiency. Furthermore, current evaluation frameworks lack flexibility in index selection, weight assignment, and adaptation to regional or institutional heterogeneity, limiting their ability to reflect the diverse performance of universities under different developmental goals [3,4].

To address these issues, it is essential to develop a comprehensive evaluation framework that accounts for both input and output efficiency while being capable of handling multi-dimensional and multi-indicator data. Data Envelopment Analysis (DEA), a non-parametric method widely used for performance evaluation, offers a promising solution. DEA can assess the relative efficiency of decision-making units (DMUs) without relying on predefined weights, making it particularly suitable for analyzing complex systems with multiple inputs and outputs [5–7]. In the context of higher education, an increasing number of studies have adopted network DEA models to unpack the internal processes of efficiency generation, thereby opening the so-called “black box” [3,8–10]. For example, Yang et al. (2018) proposed a two-stage network DDF-DEA framework to evaluate inefficiencies in key research universities under the Ministry of Education, integrating Luenberger productivity indicators for decomposition analysis [11]. Ma et al. (2022) quantitatively assessed university technology transfer efficiency across three stages—research innovation, experimental development, and value creation—in 31 Chinese universities [12]. Liu et al. (2024) evaluated the research performance of 61 Double First-Class universities using a network DEA model comprising two processes and three sub-stages [13].

Some studies have considered external and internal resource factors in evaluating university performance. Ding et al. (2023), for instance, introduced a two-stage DEA model incorporating fixed-budget constraints based on government grants [14]. Chen et al. (2023) proposed an aggregated two-stage DEA approach from the perspective of resource sharing and internal-external linkages to measure efficiency scores for 52 Chinese universities in 2014 [15]. Another strand of literature focuses on identifying key determinants of university performance across institutions [3,16,17].

Although some recent studies, e.g., [13,18,19], have acknowledged the need for group-wise comparisons, few have explicitly accounted for institutional heterogeneity in frontier construction. In practice, universities differ in aspects such as funding structures, disciplinary specializations, research infrastructure, student composition, and output profiles. Without accounting for such variations, efficiency estimates may not fully reflect actual performance differences.

In addition to methodological advances in efficiency evaluation, the second-stage analysis can also be applied to examine the determinants of efficiency through regression-based approaches. Existing studies emphasize that university performance is shaped by a combination of external environmental conditions and internal institutional characteristics [16,17,20–22]. External factors typically include the regional economic environment, the extent of governmental financial support, and policy incentives, all of which influence the resources available for academic and research activities. Internal factors, on the other hand, relate to institutional capabilities and strategic orientation, such as faculty composition and the degree of international engagement. Together, these dimensions form a comprehensive framework for understanding the drivers of efficiency variations among universities, particularly within China’s “Double First-Class” initiative, where policy priorities and institutional contexts interact in shaping performance outcomes.

Building upon these works, this study employs a two-stage network SBM DEA model to evaluate the efficiency of 67 key universities directly affiliated with the Ministry of Education in China. To address institutional heterogeneity, we adopt the meta-frontier framework proposed by [23], classifying the sample into five categories: Comprehensive Universities, Normal Universities, Medical and Pharmaceutical Universities, Science and Engineering Universities, and Agricultural and Forestry Universities [24]. By decomposing inefficiency into Technological Gap Ratio Inefficiency (TGRI) and Management Inefficiency (MI), we provide deeper insights into the sources of inefficiency across groups. The main contributions of this study are as follows:

1. **Structured efficiency assessment of Chinese research universities–**We develop a comprehensive framework that evaluates the efficiency of 67 key universities directly affiliated with the Ministry of Education through a two-stage network SBM DEA model, capturing both the R&D stage and the Technology Transfer and Application (TTA) stage.
2. **Classification-based meta-frontier analysis–**By adopting a meta-frontier framework and grouping universities into five categories (Comprehensive, Normal, Medical and Pharmaceutical, Science and Engineering, and Agricultural and Forestry), we account for institutional heterogeneity in production technology and provide more accurate group-wise efficiency benchmarks.
3. **Identification of inefficiency sources–**We decompose overall inefficiency into Technological Gap Ratio Inefficiency (TGRI) and Management Inefficiency (MI), revealing whether performance gaps are driven primarily by technological disparities across university types or by internal managerial shortcomings.

The remainder of this paper is structured as follows: Section 2 outlines the evolution of the research model and variable definitions; Section 3 presents statistical descriptions and preprocessing of variables; Section 4 reports the empirical findings, including overall efficiency, stage-specific efficiency, and inefficiency decomposition; and Section 5 concludes with policy implications and future research directions.

## 2. Method

### 2.1. Model development

Charnes et al. (1978) developed the CCR model, based on the efficiency measurement framework introduced by Farrell (1957), to evaluate the performance of DMUs with multiple inputs and multiple outputs [6,25]. Banker et al. (1984) later extended the CCR model by incorporating variable returns to scale (VRS), resulting in what is now known as the BCC model [5]. However, both of these foundational models are radial data envelopment analysis (DEA) approaches. In 2001, Tone proposed the Slacks-Based Measure (SBM) model, which differs from traditional radial models by focusing on input and output slacks directly. This non-radial approach provides a scalar efficiency score that avoids the overestimation often associated with radial DEA models [7]. Moreover, production processes are rarely as simple as a single-stage transformation. To address this limitation, Färe et al. (2007, 2014) introduced Network Data Envelopment Analysis (Network DEA), which explicitly accounts for intermediate products and internal structures, thereby opening up what was previously treated as a “black box” in conventional DEA models [8,9]. Building on this, Tone and Tsutsui (2009) proposed a weighted SBM Network DEA model that incorporates the linkages between sub-units within a DMU [26]. In this framework, each sub-unit is treated as a Sub-DMU, and the SBM approach is applied to derive optimal efficiency scores while considering interdependencies among departments. Subsequently, in 2014, Tone and Tsutsui further extended their work by integrating dynamic elements into the network structure. Based on the Dynamic SBM model proposed earlier [27], they introduced a weighted SBM Dynamic Network DEA model. This model not only considers the internal linkages among Sub-DMUs but also incorporates carry-over activities across time periods as temporal linkages [28].

**Fig 1:**
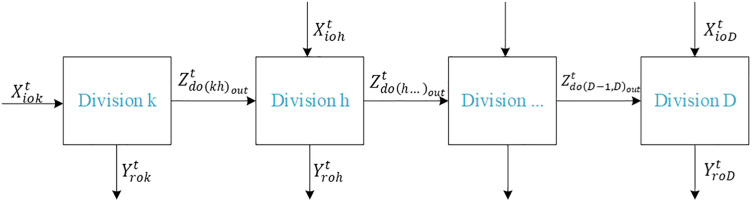
The chained network structure of the university efficiency assessment.

Furthermore, due to differences among universities in terms of funding availability, disciplinary strengths, and geographical location, their actual performance outcomes may vary significantly. Ignoring these heterogeneities could obscure the sources of inefficiency, leading to biased efficiency estimates and reducing the model’s relevance to real-world conditions. To address this issue, this study draws on the meta-frontier framework proposed by [29] and [30], which allows for a more accurate comparison across heterogeneous groups. We integrate this meta-frontier approach with a two-stage network SBM model, and further develop a chained network two-stage SBM model under the meta-frontier framework. Fig 1 shows the chained network structure of the proposed research.

### 2.2. Parameter and input-output variable settings

All universities, denoted as *N* (*j* = 1,…,*N*), are composed of *G* distinct groups of DMUs, where $\text{N}={\text{N}}_{\text{1}}+{\text{N}}_{\text{2}}+\ldots +{\text{N}}_{\text{G}}$. Under the meta-frontier framework, each DMU*p* selects the output weights that are most favorable to its own efficiency score, thereby maximizing its measured efficiency. The overall efficiency score under the common frontier can be obtained by solving the following optimization program:

Suppose there are *n* decision-making units (DMUs) (o=1,…,n); K stages (k=1,…,K); and T time periods t=1,…,T. Let $m_{k}$ and $r_{k}$ denote the number of inputs and outputs, respectively, at each stage k. The notation ${\left(k,h\right)}_{i}$ represents the link from stage k to stage h, and $L_{hk}$ denotes the set of links between divisions h and k. The definitions of inputs, outputs, and links are summarized as follows.

#### 2.2.1. Inputs and outputs.

$X_{iok}^{t}\in R_{+}(i=1,\ldots ,m_{k};\;o=1,\ldots ,n;\;k=1,\ldots ,K;\;t=1,\ldots ,T)$ refers to input i at time period t for ${DMU}_{o}$ at division k. As far as this study is concerned, in the first stage, the total number of full-time equivalent R&D personnel (in person-years) and the total R&D expenditure (in thousand yuan) are selected as the input variables. In the second stage, the number of personnel engaged in the application of R&D outcomes and science-technology services (in person-years), along with the corresponding funding allocated to these activities (in thousand yuan), are treated as additional inputs.

$Y_{rok}^{t}\in R_{+}(r=1,\ldots ,r_{k};\;o=1,\ldots ,n;\;k=1,\ldots ,K;\;t=1,\ldots ,T)$ refers to output r in time period t for ${DMU}_{o}$ at division k. The outputs from the first stage include the number of academic monographs, research articles published, software copyrights registered, patents granted, and consultation reports issued. The final outputs in the second stage consist of the number of highly cited papers, adopted consultation reports, commissioned research funding received from enterprises and institutions in the humanities and social sciences (in thousand yuan), and actual revenue generated from technology transfer (in thousand yuan).

#### 2.2.2. Links.

$Z_{do(kh{)}_{out}}^{t}\in R_{+}(d=1\ldots D;o=1,\ldots ,n;(kh)=1,\ldots ,\;{link}_{out};\;t=1,\ldots ,T$) are the period t links from ${DMU}_{o}$ from division k to division h, with (kh) being the number of k to h links; $Z_{do(kh{)}_{out}}^{t}$: the number of academic monographs, research articles published, software copyrights registered, patents granted, and consultation reports issued

**Other variables:**

$W^{k}(k=1\ldots k)$ is the weight to Division k.

### 2.3. Non-parametric two-stage meta-frontier network SBM

#### 2.3.1. Meta-frontier network SBM DEA.

Under the meta-frontier framework, each DMUp can choose the most favorable set of weights for its final outputs to maximize its own efficiency score. Consequently, the efficiency of a specific DMUp under the meta-frontier can be obtained by solving the following linear programming formulation:

Overall efficiency:


$$\begin{array}{c} \theta_{\text{p}}^{\text{meta}*}=\text{min}\frac{\sum_{\text{k}=\text{1}}^{\text{K}}{\text{W}}^{\text{k}}\left[\text{1}-\frac{\text{1}}{{\text{m}}_{\text{k}}}\left(\sum_{\text{g}=\text{1}}^{\text{G}}\sum_{\text{i}=\text{1}}^{{\text{m}}_{\text{k}}}\frac{{\text{S}}_{\text{gipk}}^{\text{t}-}}{{\text{X}}_{\text{gipk}}^{\text{t}}}\right)\right]}{\sum_{\text{k}=\text{1}}^{\text{K}}{\text{W}}^{\text{k}}\left[\text{1}+\frac{\text{1}}{{\text{r}}_{\text{k}}+{\text{link}}_{\text{k}}}\left(\sum_{\text{g}=\text{1}}^{\text{G}}\sum_{\text{r}=\text{1}}^{{\text{r}}_{\text{k}}}\frac{{\text{s}}_{\text{grpk}}^{\text{t}+}}{{\text{Y}}_{\text{grpk}}^{\text{t}}}+\;\sum_{\text{g}=\text{1}}^{\text{G}}\sum_{\left(\text{kh}\right)}^{{\text{link}}_{\text{k}}}\frac{{\text{s}}_{\text{gdp}(\text{kh})}^{\text{t}}}{{\text{Z}}_{\text{gdp}(\text{kh})}^{\text{t}}}\right)\right]} \end{array}$$ (1)

Subject to:

**Research and Development (R&D) Stage:**


$$\left\{ \begin{array}{cc} {\text{X}}_{\text{gip1}}^{\text{t}}=\sum_{\text{g}=\text{1}}^{\text{G}}\sum_{\text{o}=\text{1}}^{\text{n}}{\text{X}}_{\text{gio1}}^{\text{t}}\lambda_{\text{gio1}}^{\text{t}}+{\text{s}}_{\text{gio1}}^{\text{t}-}\left(\text{i}=\text{1},\ldots ,{\text{m}}_{\text{k}},\text{g}=\text{1}\ldots ..\text{G}\right) & \left(2.1\right)\\ {\text{Y}}_{\text{grp1}}^{\text{t}}=\sum_{\text{g}=\text{1}}^{\text{G}}\sum_{\text{o}=\text{1}}^{\text{n}}{\text{Y}}_{\text{gro1}}^{\text{t}}\lambda_{\text{gro1}}^{\text{t}}-{\text{s}}_{\text{gro1}}^{\text{t}+}\left(\text{r}=\text{1},\ldots ,{\text{r}}_{\text{k}},\text{g}=\text{1}\ldots ..\text{G}\right) & \left(2.2\right)\\ \sum_{\text{g}=\text{1}}^{\text{G}}\sum_{\text{o}=\text{1}}^{\text{n}}\lambda_{\text{k}}^{\text{t}}=\text{1}(\forall \text{k}) & \left(2.4\right)\\ \lambda_{\text{gio1}}^{\text{t}}\geq \text{0},\;\lambda_{\text{gro1}}^{\text{t}}\geq \text{0};\;{\text{s}}_{\text{gio1}}^{\text{t}-}\geq \text{0},{\text{s}}_{\text{gro1}}^{\text{t}+}\geq \text{0} & \left(2.5\right) \end{array}\right.\;$$ (2)

**Technology Transfer and Application (TTA) Stage:**


$$\left\{ \begin{array}{c} \begin{array}{cc} {\text{X}}_{\text{gip2}}^{\text{t}}=\sum_{\text{g}=\text{1}}^{\text{G}}\sum_{\text{o}=\text{1}}^{\text{n}}{\text{X}}_{\text{gio2}}^{\text{t}}\lambda_{\text{gio2}}^{\text{t}}+{\text{s}}_{\text{gio2}}^{\text{t}-}\left(\text{i}=\text{1},\ldots ,{\text{m}}_{\text{k}},\text{g}=\text{1}\ldots ..\text{G}\right) & \left(\text{3}.\text{1}\right) \end{array}\\ \begin{array}{cc} {\text{Y}}_{\text{grp2}}^{\text{t}}=\sum_{\text{g}=\text{1}}^{\text{G}}\sum_{\text{o}=\text{1}}^{\text{n}}{\text{Y}}_{\text{gro2}}^{\text{t}}\lambda_{\text{gro2}}^{\text{t}}-{\text{s}}_{\text{gro2}}^{\text{t}+}\left(\text{r}=\text{1},\ldots ,{\text{r}}_{\text{k}};\text{ g}=\text{1}\ldots ..\text{G}\right) & \left(\text{3}.\text{2}\right) \end{array}\\ \begin{array}{cc} {\text{Z}}_{\text{gdo}(\text{12})}^{\text{t}}=\sum_{\text{g}=\text{1}}^{\text{G}}\sum_{\text{o}=\text{1}}^{\text{n}}{\text{Z}}_{\text{gdo}(\text{12})}^{\text{t}}\lambda_{\text{gdo}(\text{12})}^{\text{t}}+{\text{s}}_{\text{gdo}(\text{12})}^{\text{t}}\;\left(\begin{array}{c} \text{d}=\text{1}\ldots \text{D};\text{g}=\text{1}\ldots ..\text{G};\\ \left(\text{1},\text{2})=\text{1},\text{2},\;\ldots ,({\text{link}}_{\text{out}}\right) \end{array}\right) & \left(\text{3}.\text{3}\right) \end{array}\\ \begin{array}{cc} \sum_{\text{g}=\text{1}}^{\text{G}}\sum_{\text{o}=\text{1}}^{\text{n}}\lambda_{\text{k}}^{\text{t}}=\text{1}(\forall \text{k},\;\forall \text{t}) & (3.4) \end{array}\\ \begin{array}{cc} \lambda_{\text{gio2}}^{\text{t}}\geq \text{0},\;{\;\lambda_{\text{gro2}}^{\text{t}}\geq \text{0},\;\lambda_{\text{gdo}(\text{12})}^{\text{t}}\geq \text{0};\text{s}}_{\text{gio2}}^{\text{t}-}\geq \text{0},\;{\text{ s}}_{\text{gro2}}^{\text{t}}\geq \text{0},{\text{s}}_{\text{gdo}(\text{12})}^{\text{t}}\geq \text{0} & \left(\text{3}.\text{5}\right) \end{array} \end{array}\right.\;$$ (3)

${\text{ s}}_{\text{gio1}}^{\text{t}-}\text{ and }{\text{ s}}_{\text{gro1}}^{\text{t}+}$ are stage 1 of input/output slacks. $s_{gdo(12)}^{t-}\;$is link slacks.${\text{ s}}_{\text{gio2}}^{\text{t}-}\text{ and }{\text{ s}}_{\text{gro2}}^{\text{t}+}$ are the input/output slacks at stage 2. ${\text{W}}^{\text{k}}$ indicates the weight of the two stages and it conforms to $\sum_{\text{k}=\text{1}}^{\text{K}}{\text{W}}^{\text{k}}=\text{1}$. In this study, it is believed that both stages are equally important.

#### 2.3.2. Group-frontier network SBM DEA.

Based on the following linear programming, the efficiency values for different groups are solved:


$$\begin{array}{c} \theta_{\text{p}}^{\text{group}*}=\text{min}\frac{\sum_{\text{k}=\text{1}}^{\text{K}}{\text{W}}^{\text{k}}\left[\text{1}-\frac{\text{1}}{{\text{m}}_{\text{k}}}\left(\sum_{\text{i}=\text{1}}^{{\text{m}}_{\text{k}}}\frac{{\text{S}}_{\text{ipk}}^{\text{t}-}}{{\text{X}}_{\text{ipk}}^{\text{t}}}\right)\right]}{\sum_{\text{k}=\text{1}}^{\text{K}}{\text{W}}^{\text{k}}\left[\text{1}+\frac{\text{1}}{{\text{r}}_{\text{k}}+{\text{link}}_{\text{k}}}\left(\sum_{\text{r}=\text{1}}^{{\text{r}}_{\text{k}}}\frac{{\text{s}}_{\text{rpk}}^{\text{t}+}}{{\text{Y}}_{\text{rpk}}^{\text{t}}}+\;\sum_{\left(\text{kh}\right)}^{{\text{link}}_{\text{k}}}\frac{{\text{s}}_{\text{dp}(\text{kh})}^{\text{t}}}{{\text{Z}}_{\text{dp}(\text{kh})}^{\text{t}}}\right)\right]} \end{array}$$ (4)

Subject to:

**Research and Development (R&D) Stage:**


$$\left\{ \begin{array}{cc} {\text{X}}_{\text{ip1}}^{\text{t}}=\sum_{\text{o}=\text{1}}^{\text{n}}{\text{X}}_{\text{io1}}^{\text{t}}\lambda_{\text{io1}}^{\text{t}}+{\text{s}}_{\text{io1}}^{\text{t}-}\left(\text{i}=\text{1},\ldots ,{\text{m}}_{\text{k}}\right) & \left(5.1\right)\\ {\text{Y}}_{\text{rp1}}^{\text{t}}=\sum_{\text{o}=\text{1}}^{\text{n}}{\text{Y}}_{\text{ro1}}^{\text{t}}\lambda_{\text{ro1}}^{\text{t}}-{\text{s}}_{\text{ro1}}^{\text{t}+}\left(\text{r}=\text{1},\ldots ,{\text{r}}_{\text{k}}\right) & \left(5.2\right)\\ \sum_{\text{o}=\text{1}}^{\text{n}}\lambda_{\text{k}}^{\text{t}}=\text{1}(\forall \text{k},\;\forall \text{t}) & \left(5.3\right)\\ \lambda_{\text{io1}}^{\text{t}}\geq \text{0},\;\lambda_{\text{ro1}}^{\text{t}}\geq \text{0};\;{\text{s}}_{\text{io1}}^{\text{t}-}\geq \text{0},\;{\text{s}}_{\text{ro1}}^{\text{t}+}\geq \text{0} & \left(5.4\right) \end{array}\right.\;$$ (5)

**Technology Transfer and Application (TTA) Stage:**


$$\left\{ \begin{array}{cc} {\text{X}}_{\text{ip2}}^{\text{t}}=\sum_{\text{o}=\text{1}}^{\text{n}}{\text{X}}_{\text{io2}}^{\text{t}}\lambda_{\text{io2}}^{\text{t}}+{\text{s}}_{\text{io2}}^{\text{t}-}\left(\text{i}=\text{1},\ldots ,{\text{m}}_{\text{k}}\right) & \left(6.1\right)\\ {\text{Y}}_{\text{rp2}}^{\text{t}}=\sum_{\text{o}=\text{1}}^{\text{n}}{\text{Y}}_{\text{ro2}}^{\text{t}}\lambda_{\text{ro2}}^{\text{t}}-{\text{s}}_{\text{ro2}}^{\text{t}+}\left(\text{r}=\text{1},\ldots ,{\text{r}}_{\text{k}}\right) & \left(6.2\right)\\ {\text{Z}}_{\text{do}(\text{12})}^{\text{t}}=\sum_{\text{o}=\text{1}}^{\text{n}}{\text{Z}}_{\text{do}(\text{12})}^{\text{t}}\lambda_{\text{do}(\text{12})}^{\text{t}}+{\text{s}}_{\text{do}(\text{12})}^{\text{t}}\;\left(\begin{array}{c} \text{d}=\text{1}\ldots \text{D};\\ (\text{1},\text{2})=\text{1},\text{2},\;\ldots ,\left({\text{link}}_{\text{out}}\right) \end{array}\right) & \left(6.2\right)\\ \sum_{\text{o}=\text{1}}^{\text{n}}\lambda_{\text{k}}^{\text{t}}=\text{1}(\forall \text{k},\;\forall \text{t}) & \left(6.3\right)\\ \lambda_{\text{io2}}^{\text{t}}\geq \text{0},\;{\;\lambda_{\text{ro2}}^{\text{t}}\geq \text{0},\;\lambda_{\text{do}(\text{12})}^{\text{t}}\geq \text{0};\text{s}}_{\text{io2}}^{\text{t}-}\geq \text{0},\;{\text{ s}}_{\text{ro2}}^{\text{t}+}\geq \text{0}{,\text{ s}}_{\text{do}(\text{12})}^{\text{t}}\geq \text{0} & \left(6.4\right) \end{array}\right.\;$$ (6)

### 2.4. Technology gap ratio (TGR) and inefficiency decomposition

**Definition 1:** TGR is used to measure the technological efficiency gap between the Meta-Frontier (MF) and the Group-Frontier (GF) and is seen as a measure of whether the DMU has chosen the best production technology. Since the convex front is a convex combination of each population, and in general, the efficiency obtained under MF is smaller than that under GF, there will be an MFE less than the GFE. It also means that 0<TGR≤1. TGR can be calculated by the following formula:


$$\begin{array}{c} \text{MTGR}=\frac{\text{MFE}}{\text{GFE}} \end{array}$$ (7)

**Definition 2:** In addition, in order to explore the efficiency at MF and GF respectively and as well as the policy implications behind their formation, we referred to the methodology of [23,27,31]. DMU inefficiency under MF can be broken down into Technological Gap Ratio Inefficiency (TGRI) at the frontier of a specific group and Management Inefficiency under the specific group (Group Managerial Inefficiency, GMI). TGRI represents the inefficiency of the DMUp at the group-specific frontier, which stems from the technological gap between meta-frontier and group-specific frontier. The specific formula is as follows:


$$\begin{array}{c} \text{TGRI}=\text{GFE}*(\text{1}-\text{MTGR}) \end{array}$$ (8)

**Definition 3**: Group Managerial Inefficiency (GMI) represents the inefficiency of DMUs in a specific group frontier. The reason for the inefficiency is attributed to the management failure of DMUs. Its calculation formula is as follows:


$$\begin{array}{c} \text{GMI}=\text{1}-\text{GFE} \end{array}$$ (9)

Meanwhile, the inefficiency based on the meta-frontier can be expressed as


$$\begin{array}{c} \text{MTI}=\text{TGRI}+\text{GMI} \end{array}$$ (10)

## 3. Statistical description, data processing and source

### 3.1. Statistical description of input, intermediate and output variables

Table 1 shows the statistical description of the input, output and intermediate variables at the R&D stage and TTA stage respectively.

**Table 1:** Statistical description of input, intermediate and output variables.

Stage	Variable		Maximum	Minimum	Mean	S.D.
Research and Development (R&D) Stage	Input	Total Number of R&D Personnel (Person-Year)	6833	212	1344.94	948.28
		Total R&D Expenditure (Thousand Yuan)	4171270	10815	78099	666719
	Output (intermediate)	Number of Think Tank Reports (Article)	702	1	80	110.17
		Number of Publications (Articles)	17025	1142	6439.94	3727.8
		Number of Patents and Software Copyrights (Item)	3232	4	908.53	634.6
Technology Transfer and Application (TTA) Stage	Additional input	R&D Achievement Transformation Personnel (Person-Year)	690	2	176.44	147.95
		R&D Achievement Transformation Expenditure (Thousand Yuan)	1487074	299	152894	209029
	Output	Number of Highly Cited Papers (Article)	396	3	72.79	69.77
		Number of Consultation Reports Adopted (Article)	646	1	54.19	78.57
		Enterprise/Institutional Commissioned Funding in Humanities and Social Sciences (Thousand Yuan)	197030	99	27058	37032.6
		Technology Transfer Revenue (Thousand Yuan)	311100	82	25378	48930.2

### 3.2. Data processing and source

In analyzing the transformation of scientific and technological achievements into practical applications, it is crucial to consider the inherent time lags between research inputs and their resulting outputs. Moreover, considering that universities require time to conduct research and innovation, experimental development, and value creation activities, this study adopts a one-year lag to better capture the temporal delay between research inputs and their realized outputs. Consistent with methodologies used in previous studies [12,32,33], we apply a one-year lag to the measurement of output indicators. Specifically, input data such as R&D investment correspond to year t, while intermediate outputs (e.g., research outcomes) and final outputs related to technology transfer are taken from years t+1 and t+2, respectively. For this study, input data on technology transfer activities cover the period from 2014 to 2018; outcome indicators reflecting scientific achievements are drawn from 2015 to 2019; and data on actual technology commercialization span from 2016 to 2020.

The majority of the statistical data used in this study are obtained from the *Data Analysis Report and Compilation of Basic Information on Teaching and Education in Universities Directly Affiliated with the Ministry of Education* (2013–2020). The dataset includes various indicators such as R&D funding and personnel, funding and staffing for the application of R&D outcomes and science-technology services, number of scientific publications and monographs, submission and adoption rates of think tank reports, number of granted patents, value of signed technology transfer contracts, and commissioned research funding from enterprises and institutions. In addition, data on software copyrights are sourced from the Tianyancha database (www.tianyancha.com). Based on the registration dates, we compiled the annual number of software copyright registrations for each university. Citation-based indicators such as total citation counts, average citations per paper, number of highly cited papers, field-normalized impact scores, proportion of highly cited papers, and H-index are derived from Clarivate Analytics’ InCites database, which is built upon authoritative citation data from Web of Science (including SCIE, SSCI, and A&HCI).

## 4. Empirical analysis

### 4.1. Overall efficiency analysis

**Fig 2:**
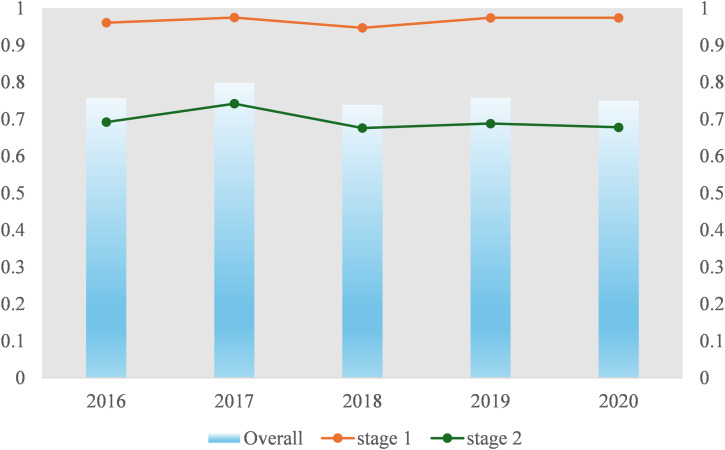
Overall, R&D Stage and TTA Stage efficiencies.

Overall, the comprehensive efficiency of the sampled universities remained relatively stable between 2016 and 2020 (Fig 2). While the efficiency scores exhibited a fluctuating pattern—first increasing, then decreasing, and subsequently rising again—they generally stayed within the range of 0.7 to 0.8, indicating room for improvement in overall performance. The efficiency scores of the R&D stage remained consistently high, exceeding 0.9 throughout the five-year period, suggesting that these universities were operating very close to the production frontier in terms of research input utilization.

In contrast, the efficiency of the technology transfer and application (TTA) stage followed a trend of initial increase, followed by a decline, and eventual stabilization at around 0.7. This relatively low level of efficiency highlights a key challenge in the innovation process: although research inputs are being effectively converted into academic outputs, the subsequent transformation of these outputs into practical applications remains inefficient.

Taken together, these findings indicate that the sampled universities did not achieve full efficiency during the study period, primarily due to inefficiencies in the TTA stage. This further confirms the prevailing tendency in Chinese higher education institutions to emphasize research output over the practical application of scientific achievements—what can be described as a pattern of “valuing research output while neglecting technology transfer.” As a result, the effective commercialization and societal impact of technological innovations have yet to receive sufficient attention and development.

### 4.2. Overall, stage and TGR analysis of different universities

As shown in Table 2, the average overall efficiency scores as well as the stage-specific efficiencies for the R&D and Technology Transfer and Application (TTA) stages were calculated for all sampled universities over the period 2016–2020. Specifically, thirteen universities—including BUAA, BUCT, BNU, BUCM, CHU, HHU, NCEPU, CCNU, NJU, SNNU, OUC, USTC, and RUC—achieved optimal efficiency scores of 1 across both comprehensive and two-stage evaluations, indicating that they operated on the best-practice frontier. Among these efficient institutions, three are comprehensive universities (PKU, FDU, XMU), six are science and engineering universities (BUAA, BUCT, CHU, HHU, USTC, and RUC), three are normal universities (BNU, CCNU, and SNNU), and one is a medical university (BUCM).

**Table 2:** Overall efficiency and two stages efficiencies under meta-frontier.

University	Overall efficiency	Rank 1	R&D stage	Rank 2	TTA stage	Rank 3	University	Overall efficiency	Rank 1	R&D stage			Rank 2	TTA stage	Rank 3	University	Overall efficiency	Rank 1	R&D stage	Rank 2	TTA stage	Rank 3
PKU	0.780	37	**1.000**	1	0.706	1	HNU	0.877	26	**1.000**			1	0.836	23	TianJU	0.556	53	**1.000**	1	0.407	45
BUAA	**1.000**	1	**1.000**	1	**1.000**	1	NCEPU	**1.000**	1	**1.000**			1	**1.000**	24	TongJU	0.475	57	0.984	46	0.306	46
BUCT	**1.000**	1	**1.000**	1	**1.000**	1	ECUST	0.695	45	**1.000**			1	0.594	25	WHU	0.923	21	**1.000**	1	0.897	47
BJTU	0.314	67	0.827	64	0.149	1	ECNU	0.890	23	**1.000**			1	0.853	26	WHUT	0.925	20	**1.000**	1	0.900	48
USTB	0.343	66	0.844	60	0.185	1	SCUT	0.970	15	**1.000**			1	0.961	27	XDU	0.639	48	0.927	55	0.551	49
BIT	0.834	32	0.938	53	0.793	1	HUST	0.732	41	**1.000**			1	0.643	28	XJTU	0.851	30	**1.000**	1	0.801	50
BFU	0.879	25	**1.000**	1	0.839	1	HZAU	0.577	51	0.953			51	0.455	29	NPU	0.868	27	0.970	49	0.838	51
BNU	**1.000**	1	**1.000**	1	**1.000**	1	CCNU	**1.000**	1	**1.000**			1	**1.000**	30	NWAFU	0.445	59	0.989	45	0.265	52
BUPT	0.377	64	0.974	48	0.176	1	JLU	0.688	47	0.896			58	0.631	31	SWU	0.945	18	**1.000**	1	0.927	53
BUCM	**1.000**	1	**1.000**	1	**1.000**	1	JNU	0.951	16	**1.000**			1	0.934	32	SWJTU	0.756	40	0.999	43	0.675	54
DUT	0.441	60	0.956	50	0.273	1	LZU	0.912	22	**1.000**			1	0.883	33	CHD	**1.000**	1	**1.000**	1	**1.000**	55
UESTC	0.618	50	0.834	62	0.555	1	NJU	**1.000**	1	**1.000**			1	**1.000**	34	ZJU	0.690	46	**1.000**	1	0.587	56
NEU	0.721	43	**1.000**	1	0.628	1	NUAA	0.698	44	**1.000**			1	0.598	35	CUG	0.453	58	0.841	61	0.337	57
NEFU	0.511	54	0.941	52	0.372	14	NJUST	0.779	38	**1.000**			1	0.706	36	OUC	**1.000**	1	**1.000**	1	**1.000**	58
NENU	0.834	33	**1.000**	1	0.779	15	NJAU	0.494	55	0.920			56	0.364	37	USTC	**1.000**	1	**1.000**	1	**1.000**	59
DHU	0.774	39	**1.000**	1	0.698	16	NKU	0.994	14	**1.000**			1	0.992	38	CUMT	0.825	34	**1.000**	1	0.767	60
SEU	0.624	49	0.918	57	0.531	17	THU	0.867	28	**1.000**			1	0.823	39	CAU	0.427	61	0.929	54	0.269	61
FDU	0.864	29	**1.000**	1	0.819	18	XMU	0.806	35	**1.000**			1	0.742	40	RUC	**1.000**	1	**1.000**	1	**1.000**	62
HEU	0.568	52	0.823	65	0.499	19	SDU	0.724	42	0.976			47	0.644	41	CUP	0.370	65	0.696	67	0.290	63
HIT	0.402	62	0.822	66	0.270	20	SNNU	**1.000**	1	**1.000**			1	**1.000**	42	CPU	0.946	17	**1.000**	1	0.928	64
HFUT	0.488	56	0.857	59	0.372	21	SJTU	0.928	19	**1.000**			1	0.904	43	CSU	0.796	36	0.999	43	0.729	65
HHU	**1.000**	1	**1.000**	1	**1.000**	22	SCU	0.391	63	0.828			63	0.264	44	SYSU	0.841	31	**1.000**	1	0.787	66
																CQU	0.881	24	**1.000**	1	0.841	67
Comprehensive Universities				0.822/0.984/0.769			Normal Universities				0.945/1.000/0.926				Medical and Pharmaceutical Universities				0.973/1.000/0.964			
Science and Engineering Universities				0.704/0.944/0.628			Agricultural and Forestry Universities					0.556/0.955/0.427			Overall average				0.761/0.966/0.695			

In contrast, ten universities—including TongJU, CUP, DUT, HIT, CAU, NWAFU, SCU, USTB, BUPT, and BJTU—exhibited generally low levels of comprehensive and TTA stage efficiency. Among them, two are comprehensive universities (TongJU, SCU), six are science and engineering universities (CUP, DUT, HIT, USTB, BUPT, BJTU), and two are agricultural and forestry universities (CAU, NWAFU).

Further analysis of input excesses and output shortfalls among these inefficient universities reveals the following patterns: BJTU, BUPT, SCU, and CAU show output deficiencies primarily in terms of academic publications and monographs, as well as think tank reports. USTB, CUP, DUT, and NWAFU exhibit significant output shortfalls across multiple indicators, including publications, monographs, think tank reports, adopted policy reports, technology transfer revenue, and commissioned research funding from enterprises and institutions. Finally, HIT and TongJU display both input overuse and output underperformance, particularly in terms of excessive R&D expenditure and insufficient levels of commissioned research funding and adopted think tank reports.

There are significant differences in the efficiency of technology transfer among universities under the meta-frontier and group-specific frontiers. As shown in Table 3: First, the efficiency of technology transfer under the meta-frontier is notably lower than that under the group-specific frontier. Over the period 2016–2020, the average technology transfer efficiency under the meta-frontier was 0.761, compared to 0.905 under the group frontier. A similar pattern is observed for both the output stage and the overall efficiency. Second, across both frontiers, the efficiency of the technology transfer stage is consistently lower than that of the output stage—consistent with earlier findings. Specifically, the average output stage efficiencies under the meta-frontier and group frontier were 0.966 and 0.981, respectively, whereas the corresponding technology transfer stage efficiencies were significantly lower at 0.695 and 0.883. Despite multiple national policies and financial incentives aimed at promoting technology transfer, its efficiency remains below expectations. Third, there are substantial variations in technology transfer performance across different types of universities. Under the meta-frontier framework, all university categories generally exhibit the following ranking: output stage efficiency > comprehensive efficiency > technology transfer stage efficiency. In terms of comprehensive and technology transfer stage efficiencies, the ranking is as follows: medical and pharmaceutical universities > normal universities > comprehensive universities > science and engineering universities > agricultural and forestry universities. In terms of output stage efficiency, the order is: medical and pharmaceutical universities = normal universities > comprehensive universities > agricultural and forestry universities > science and engineering universities.

**Table 3:** Overall, R&D stage and TTA stage efficiency under meta and group frontiers.

University	Overall			R&D Stage			TTA Stage			University	Overall			R&D Stage			TTA Stage			University	Overall			R&D Stage			TTA Stage		
	Meta	Group	TGR	Meta	Group	TGR	Meta	Group	TGR		Meta	Group	TGR	Meta	Group	TGR	Meta	Group	TGR		Meta	Group	TGR	Meta	Group	TGR	Meta	Group	TGR
PKU	0.780	0.937	0.840	**1.000**	**1.000**	**1.000**	0.706	0.917	0.785	HNU	0.877	0.910	0.948	**1.000**	**1.000**	**1.000**	0.836	0.880	0.911	TianJU	0.556	0.675	0.845	**1.000**	**1.000**	**1.000**	0.407	0.566	0.768
BUAA	**1.000**	**1.000**	**1.000**	**1.000**	**1.000**	**1.000**	**1.000**	**1.000**	**1.000**	NCEPU	**1.000**	**1.000**	**1.000**	**1.000**	**1.000**	**1.000**	**1.000**	**1.000**	**1.000**	TongJU	0.475	0.650	0.758	0.984	0.992	0.992	0.306	0.536	0.593
BUCT	**1.000**	**1.000**	**1.000**	**1.000**	**1.000**	**1.000**	**1.000**	**1.000**	**1.000**	ECUST	0.695	0.962	0.714	**1.000**	**1.000**	**1.000**	0.594	0.950	0.607	WHU	0.923	**1.000**	0.923	**1.000**	**1.000**	**1.000**	0.897	**1.000**	0.897
BJTU	0.314	**1.000**	0.314	0.827	**1.000**	0.827	0.149	1.000	0.149	ECNU	0.890	0.968	0.917	**1.000**	**1.000**	**1.000**	0.853	0.957	0.886	WHUT	0.925	0.971	0.946	**1.000**	**1.000**	**1.000**	0.900	0.962	0.923
USTB	0.343	0.603	0.602	0.844	0.847	0.996	0.185	0.550	0.388	SCUT	0.970	**1.000**	0.970	**1.000**	**1.000**	**1.000**	0.961	**1.000**	0.961	XDU	0.639	0.774	0.840	0.927	0.989	0.939	0.551	0.705	0.826
BIT	0.834	0.871	0.951	0.938	0.973	0.964	0.793	0.869	0.912	HUST	0.732	0.898	0.816	**1.000**	**1.000**	**1.000**	0.643	0.864	0.745	XJTU	0.851	**1.000**	0.851	**1.000**	**1.000**	**1.000**	0.801	**1.000**	0.801
BFU	0.879	**1.000**	0.879	**1.000**	**1.000**	**1.000**	0.839	**1.000**	0.839	HZAU	0.577	**1.000**	0.577	0.953	**1.000**	0.953	0.455	**1.000**	0.455	NPU	0.868	0.967	0.887	0.970	**1.000**	0.970	0.838	0.957	0.860
BNU	**1.000**	**1.000**	**1.000**	**1.000**	**1.000**	**1.000**	**1.000**	**1.000**	**1.000**	CCNU	**1.000**	**1.000**	**1.000**	**1.000**	**1.000**	**1.000**	**1.000**	**1.000**	**1.000**	NWAFU	0.445	**1.000**	0.445	0.989	**1.000**	0.989	0.265	**1.000**	0.265
BUPT	0.377	0.676	0.580	0.974	0.984	0.990	0.176	0.580	0.307	JLU	0.688	0.895	0.784	0.896	0.942	0.962	0.631	0.888	0.717	SWU	0.945	**1.000**	0.945	**1.000**	**1.000**	**1.000**	0.927	**1.000**	0.927
BUCM	**1.000**	**1.000**	**1.000**	**1.000**	**1.000**	**1.000**	**1.000**	**1.000**	**1.000**	JNU	0.951	**1.000**	0.951	**1.000**	**1.000**	**1.000**	0.934	**1.000**	0.934	SWJTU	0.756	**1.000**	0.756	0.999	**1.000**	0.999	0.675	**1.000**	0.675
DUT	0.441	0.562	0.802	0.956	0.972	0.984	0.273	0.428	0.660	LZU	0.912	**1.000**	0.912	**1.000**	**1.000**	**1.000**	0.883	**1.000**	0.883	CHD	**1.000**	**1.000**	**1.000**	**1.000**	**1.000**	**1.000**	**1.000**	**1.000**	**1.000**
UESTC	0.618	0.860	0.714	0.834	0.867	0.962	0.555	0.912	0.590	NJU	**1.000**	**1.000**	**1.000**	**1.000**	**1.000**	**1.000**	**1.000**	**1.000**	**1.000**	ZJU	0.690	0.993	0.694	**1.000**	**1.000**	**1.000**	0.587	0.990	0.590
NEU	0.721	0.821	0.868	**1.000**	**1.000**	**1.000**	0.628	0.762	0.797	NUAA	0.698	0.826	0.847	**1.000**	**1.000**	**1.000**	0.598	0.768	0.778	CUG	0.453	0.813	0.561	0.841	0.898	0.943	0.337	0.807	0.420
NEFU	0.511	**1.000**	0.511	0.941	**1.000**	0.941	0.372	1.000	0.372	NJUST	0.779	0.956	0.806	**1.000**	**1.000**	**1.000**	0.706	0.942	0.730	OUC	**1.000**	**1.000**	**1.000**	**1.000**	**1.000**	**1.000**	**1.000**	**1.000**	**1.000**
NENU	0.834	0.892	0.924	**1.000**	**1.000**	**1.000**	0.779	0.856	0.872	NJAU	0.494	**1.000**	0.494	0.920	**1.000**	0.920	0.364	**1.000**	0.364	USTC	**1.000**	**1.000**	**1.000**	**1.000**	**1.000**	**1.000**	**1.000**	**1.000**	**1.000**
DHU	0.774	**1.000**	0.774	**1.000**	**1.000**	**1.000**	0.698	**1.000**	0.698	NKU	0.994	**1.000**	0.994	**1.000**	**1.000**	**1.000**	0.992	**1.000**	0.992	CUMT	0.825	0.901	0.900	**1.000**	**1.000**	**1.000**	0.767	0.868	0.836
SEU	0.624	0.813	0.735	0.918	**1.000**	0.918	0.531	0.751	0.653	THU	0.867	**1.000**	0.867	**1.000**	**1.000**	**1.000**	0.823	**1.000**	0.823	CAU	0.427	**1.000**	0.427	0.929	**1.000**	0.929	0.269	1.000	0.269
FDU	0.864	0.994	0.868	**1.000**	**1.000**	**1.000**	0.819	0.991	0.823	XMU	0.806	0.960	0.834	**1.000**	**1.000**	**1.000**	0.742	0.947	0.773	RUC	**1.000**	**1.000**	**1.000**	**1.000**	**1.000**	**1.000**	**1.000**	**1.000**	**1.000**
HEU	0.568	0.610	0.958	0.823	0.823	**1.000**	0.499	0.554	0.940	SDU	0.724	**1.000**	0.724	0.976	**1.000**	0.976	0.644	1.000	0.644	CUP	0.370	0.523	0.735	0.696	0.712	1.032	0.290	0.523	0.689
HIT	0.402	0.453	0.916	0.822	0.882	0.932	0.270	0.313	0.950	SNNU	**1.000**	**1.000**	**1.000**	**1.000**	**1.000**	**1.000**	**1.000**	**1.000**	**1.000**	CPU	0.946	**1.000**	0.946	**1.000**	**1.000**	**1.000**	0.928	**1.000**	0.928
HFUT	0.488	0.586	0.796	0.857	0.943	0.903	0.372	0.471	0.703	SJTU	0.928	0.964	0.960	**1.000**	**1.000**	**1.000**	0.904	0.952	0.943	CSU	0.796	0.915	0.850	0.999	**1.000**	0.999	0.729	0.886	0.769
HHU	**1.000**	**1.000**	**1.000**	**1.000**	**1.000**	**1.000**	**1.000**	**1.000**	**1.000**	SCU	0.391	0.543	0.762	0.828	0.970	0.852	0.264	0.404	0.704	SYSU	0.841	0.912	0.917	**1.000**	**1.000**	**1.000**	0.787	0.882	0.876
																				CQU	0.881	**1.000**	0.881	**1.000**	**1.000**	**1.000**	0.841	**1.000**	0.841

Notably, normal and medical universities achieved average annual efficiency scores above 0.9 across all stages, indicating strong overall performance. In contrast, comprehensive, science and engineering, and agricultural and forestry universities recorded average comprehensive and technology transfer stage efficiencies below 0.85, although their output stage efficiencies remained mostly above 0.95. This suggests that while these institutions perform well in generating research outputs, they lag behind in effectively translating those outputs into practical applications.

In addition, the average technological gap ratio (TGR) across all universities from 2016 to 2020 was 0.836, close to 1, indicating a relatively small technological gap between the group frontier and the meta-frontier on the whole. However, considerable heterogeneity exists across individual universities. On one hand, TGR values vary significantly by institutional type. Among the sampled universities, 13 achieved a TGR of 1, including six science and engineering universities, three normal universities, three comprehensive universities, and one medical university. Conversely, the five lowest-performing universities in terms of TGR were primarily composed of four agricultural and forestry universities and one science and engineering university, highlighting clear inter-type disparities. On the other hand, from a temporal perspective, the TGR increased from 0.848 in 2016 to 0.867 in 2020, indicating an overall narrowing of the technological gap over time. Specifically, normal and medical universities showed a steady increase followed by stabilization, suggesting minimal technological gaps within these groups. In contrast, comprehensive, science and engineering, and agricultural and forestry universities exhibited an initial decline followed by recovery—indicating a temporary widening of the technological gap before it began to narrow again. Nonetheless, agricultural and forestry universities still face the largest technological gap compared to other types of institutions.

### 4.3. Meta-frontier decomposition and inefficiency analysis

#### 4.3.1. Four quadrant analysis of inefficient sources.

In the process of technology transfer at universities, efficiency loss (Efficiency Loss, EL) can be decomposed into technological gap inefficiency (TGRI) and management inefficiency (MI). Specifically, TGRI is calculated as the difference between the group-specific frontier and the meta-frontier efficiency scores, while MI reflects the gap between each university’s performance under the group frontier and its potential optimal performance on the production frontier.

**Fig 3:**
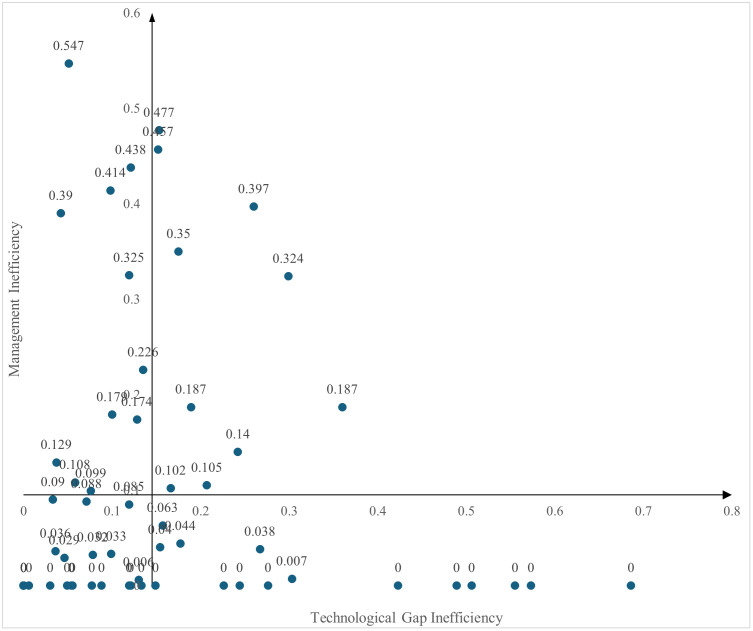
Four quadrant diagram of management inefficiency and technology gap inefficiency.

By decomposing the sources of inefficiency and visualizing the results in a quadrant plot (Fig 3), this study classifies universities based on whether their average TGRI and MI values are above or below the sample mean. The classification yields four categories: (1) high-TGRI and high-MI, (2) low-TGRI and high-MI, (3) low-TGRI and low-MI, and (4) high-TGRI and low-MI.

- Quadrant I (High TGRI & High MI): This group includes ten universities such as USTB, BUPT, and UESTC, accounting for 14.9% of the total sample. These are primarily comprehensive and science and engineering universities that exhibit weaknesses in both technological capability and managerial effectiveness.
- Quadrant II (Low TGRI & High MI): This group comprises eleven universities including BIT, DUT, and NEU, representing 16.4% of the sample. Most of them are science and engineering universities, characterized by relatively small technological gaps but significant management inefficiencies.
- Quadrant III (Low TGRI & Low MI): The largest group, with 31 universities (46.3% of the sample), includes institutions such as BUAA, BUCT, and BFU. These universities perform well in both dimensions and are predominantly comprehensive universities, followed by science and engineering, normal, medical, and agricultural and forestry universities.
- Quadrant IV (High TGRI & Low MI): This group contains fifteen universities such as PKU, BJTU, and NEFU, making up 22.4% of the total. These universities show relatively strong management capabilities but face notable technological limitations. The types of universities in this group are more evenly distributed, with five each from the comprehensive, science and engineering, and agricultural and forestry categories.

Overall, the quadrant analysis reveals distinct patterns of efficiency loss among universities in the context of technology transfer. These findings suggest that targeted strategies—such as addressing technological constraints or improving management practices—are necessary to enhance overall technology transfer performance across different types of institutions.

#### 4.3.2. Stage analysis of inefficiency source.

Based on the decomposition of inefficiency into technological gap inefficiency and managerial inefficiency as discussed earlier, Fig 3 presents the efficiency loss of the sampled universities across the comprehensive stage, output stage, and technology transfer stage. It also illustrates the percentage contribution of each source of inefficiency—technological versus managerial—to the overall efficiency loss. Detailed results are reported in Table 4.

**Table 4:** Inefficient decomposition of R&D stage and TTA stage.

DMU	Overall			R&D stage			TTA stage			DMU	Overall			R&D stage			TTA stage		
	Inefficiency value	TGRI	MI		Inefficiency value	TGRI	MI	Inefficiency value	TGRI	MI	Inefficiency value	TGRI	MI	Inefficiency value	TGRI	MI	Inefficiency value	TGRI	MI
PKU	0.220	71%	29%	–	–	–	0.294	72%	28%	LZU	–	–	–	–	–	–	0.117	100%	–
BUAA	–	–	–	–	–	–	–	–	–	NJU	–	–	–	–	–	–	–	–	–
BUCT	–	–	–	–	–	–	–	–	–	NUAA	0.302	42%	58%	–	–	–	0.402	42%	58%
BJTU	0.686	100%	–	0.173	100%	–	0.851	100%	–	NJUST	0.221	80%	20%	–	–	–	0.294	80%	20%
USTB	0.657	40%	60%	0.156	2%	98%	0.815	45%	55%	NJAU	0.506	100%	–	0.080	100%	–	0.636	100%	–
BIT	0.166	22%	78%	0.062	56%	44%	0.207	37%	63%	NKU	–	–	–	–	–	–	0.008	100%	–
BFU	–	–	–	–	–	–	0.161	100%	–	THU	0.133	100%	–	–	–	–	0.177	100%	–
BNU	–	–	–	–	–	–	–	–	–	SDU	0.276	100%	–	0.024	100%	–	0.356	100%	–
BUPT	0.623	48%	52%	0.026	38%	62%	0.824	49%	51%	SNNU	–	–	–	–	–	–	–	–	–
BUCM	–	–	–	–	–	–	–	–	–	SJTU	0.072	50%	50%	–	–	–	0.096	50%	50%
CHU	–	–	–	–	–	–	–	–	–	SCU	0.609	25%	75%	0.172	83%	17%	0.736	19%	81%
DUT	0.559	22%	78%	0.044	36%	64%	0.727	21%	79%	TianJU	0.444	27%	73%	–	–	–	0.593	27%	73%
UESTC	0.382	63%	37%	0.166	20%	80%	0.445	80%	20%	TongJU	0.525	33%	67%	0.016	50%	50%	0.694	33%	67%
NEU	0.279	36%	64%	–	–	–	0.372	36%	64%	WHU	–	–	–	–	–	–	0.103	100%	–
NEFU	0.489	100%	–	0.059	100%	–	0.628	100%	–	WHUT	0.075	61%	39%	–	–	–	0.100	62%	38%
NENU	0.166	35%	65%	–	–	–	0.221	35%	65%	XDU	0.361	37%	63%	0.073	85%	15%	0.449	34%	66%
DHU	0.226	100%	–	–	–	–	0.302	100%	–	XJTU	–	–	–	–	–	–	0.199	100%	–
SEU	0.376	50%	50%	0.082	100%	–	0.469	47%	53%	NPU	0.132	75%	25%	0.030	100%	–	0.162	73%	27%
FDU	–	–	–	–	–	–	0.181	95%	5%	NWAFU	0.555	100%	–	0.011	100%	–	0.735	100%	–
HEU	0.432	10%	90%	0.177	–	100%	0.501	11%	89%	SWU	–	–	–	–	–	–	0.073	100%	–
HIT	0.598	9%	91%	0.178	34%	66%	0.730	6%	94%	SWJTU	–	–	–	0.001	100%	–	0.325	100%	–
HFUT	0.512	19%	81%	0.143	60%	40%	0.628	16%	84%	XMU	0.194	79%	21%	–	–	–	0.258	79%	21%
HHU	–	–	–	–	–	–	–	–	–	ZJU	0.310	98%	2%	–	–	–	0.413	98%	2%
HNU	0.123	27%	73%	–	–	–	0.164	27%	73%	CUG	0.547	66%	34%	0.159	36%	64%	0.663	71%	29%
NCEPU	–	–	–	–	–	–	–	–	–	OUC	–	–	–	–	–	–	–	–	–
ECUST	0.305	88%	12%	–	–	–	0.406	88%	12%	USTC	–	–	–	–	–	–	–	–	–
ECNU	0.110	71%	29%	–	–	–	0.147	71%	29%	CUMT	–	–	–	–	–	–	0.233	43%	57%
SCUT	–	–	–	–	–	–	0.039	100%	–	CAU	0.573	100%	–	0.071	100%	–	0.731	100%	–
HUST	0.268	62%	38%	–	–	–	0.357	62%	38%	RUC	–	–	–	–	–	–	–	–	–
HZAU	0.423	100%	–	0.047	100%	–	0.545	100%	–	CUP	0.630	24%	76%	0.304	5%	95%	0.710	33%	67%
CCNU	–	–	–	–	–	–	–	–	–	CPU	–	–	–	–	–	–	0.072	100%	–
JLU	0.312	66%	34%	0.104	44%	56%	0.369	70%	30%	CSU	0.204	58%	42%	0.001	100%	–	0.271	58%	42%
JNU	0.049	100%	–	–	–	–	0.066	100%	–	SYSU	0.159	45%	55%	–	–	–	0.213	45%	55%
										CQU	–	–	–	–	–	–	0.159	100%	–

Overall, a total of 25 universities operate on the best-practice frontier and exhibit no efficiency loss. However, 24 universities experience significant efficiency losses due to technological gap inefficiency (TGRI), with half of them showing TGRI accounting for more than 85% of total inefficiency. Additionally, 17 universities suffer from substantial management inefficiency (MI), among which two universities have MI contributing over 85% to their overall efficiency loss. In the R&D stage, 42 universities are located on the production frontier and thus achieve full efficiency. In contrast, 15 universities face major efficiency losses due to TGRI, with most of these institutions reporting TGRI shares exceeding 85%. Nine universities show high levels of MI, and among them, three have MI contributing more than 85% to their total inefficiency. In TTA stage, only 13 universities perform at the optimal level. A large number of universities—34 in total—experience significant efficiency losses due to technological gaps, with 24 of them having TGRI contributions above 85%. Meanwhile, 20 universities are affected by notable management inefficiencies, and in two cases, MI accounts for over 85% of total inefficiency.

Overall, technological differences contribute more significantly to efficiency loss than management inefficiencies across all stages and university types. Moreover, the majority of both TGRI and MI stem from the TTA stage, highlighting it as the primary source of inefficiency in the technology transfer process. Take Beijing Jiaotong University as an example: its overall efficiency loss in the comprehensive stage is 0.686, entirely attributed to technological gaps. While the efficiency loss in the R&D stage is relatively small (0.173), the TTA stage exhibits a much higher loss (0.851), indicating that this stage is the main driver of inefficiency.

Another representative case is Southeast University. Its comprehensive efficiency loss is 0.376, equally divided between TGRI and MI. In the R&D stage, the efficiency loss is minimal (0.082) and fully attributable to technological gaps. However, in the TTA stage, the efficiency loss increases to 0.469, with 47% due to TGRI and 53% due to MI. This suggests that while SEU faces technological limitations in both the output and transfer stages, its management inefficiencies are primarily concentrated in the TTA stage.

These findings indicate that different universities should adopt targeted strategies based on their specific sources of inefficiency: For universities where technological gaps are the dominant source of inefficiency, efforts should focus on upgrading research infrastructure, modernizing laboratory equipment, attracting high-caliber researchers, and strengthening collaborations with enterprises and research institutions to enhance innovation capabilities. For universities suffering from management inefficiencies, improvements should be made in administrative processes, including the adoption of modern management practices, enhancement of internal monitoring systems, and implementation of performance-based evaluation mechanisms to ensure efficient resource allocation and utilization.

## 5. Finding and recommendation

### 5.1. Key findings

This study evaluates the efficiency of technology transfer among Chinese universities directly affiliated with the Ministry of Education from 2016 to 2020, using a combined meta-frontier and network SBM DEA approach. By analyzing overall efficiency, stage-specific efficiency, and sources of inefficiency, we provide a comprehensive assessment of the performance of university-based technology transfer in China.

The key findings are as follows: 1) The overall efficiency of technology transfer is at a relatively high level, with some universities achieving full efficiency by operating on the best-practice frontier. However, notable discrepancies exist between group-specific frontiers and the meta-frontier. In particular, the efficiency of TTA stage is significantly lower than that of the R&D stage, indicating that the transformation of research outputs into practical applications remains a weak link in the innovation chain. 2) Significant heterogeneity exists across university types. Normal universities and medical & pharmaceutical universities consistently perform well, with average annual efficiency scores exceeding 0.9 across all stages. In contrast, comprehensive, science and engineering, and agricultural and forestry universities exhibit lower efficiency—particularly in the TTA stage, where efficiency values fall below 0.85, suggesting the need for improved resource allocation and more effective technology transfer mechanisms. 3) Technological gaps are the primary source of inefficiency. Technological gap-related inefficiency (TGRI) accounts for a larger proportion of total inefficiency than management inefficiency (MI), especially in the TTA stage. Further decomposition under the meta-frontier framework and quadrant analysis reveal that most universities fall into the “low TGRI–low MI” category. However, a number of institutions remain in the “high TGRI–high MI” quadrant, indicating dual disadvantages in both technological capability and managerial effectiveness. Overall, the TTA stage exhibits more pronounced efficiency losses due to both TGRI and MI, compared to the R&D stage. Case studies of Beijing Jiaotong University and Southeast University illustrate the coexistence of technological limitations and managerial shortcomings in certain institutions.

### 5.2. Policy recommendations

Based on the empirical findings, this study proposes the following policy recommendations to enhance the efficiency of technology transfer in Chinese higher education institutions:

1 **Addressing technological gaps**

Technological gaps are identified as the dominant source of inefficiency, particularly in the Technology Transfer and Application (TTA) stage. To bridge these gaps, universities should prioritize upgrading scientific infrastructure, including modern laboratory equipment, maintenance systems, and research facilities. Recruiting high-caliber researchers and strengthening collaborations with enterprises and research institutions can further enhance innovation capabilities. Moreover, fostering industry-university-research partnerships and accelerating the commercialization of research outcomes will help close the gap between academic research and industrial application.

2 **Improving management efficiency**

Management inefficiencies, especially in the TTA stage, indicate the need for institutional reforms. Universities should streamline technology transfer processes, minimize bureaucratic procedures, and remove unnecessary intermediaries to improve operational efficiency. Introducing modern management practices, establishing robust internal monitoring systems, and implementing performance-based evaluation frameworks can ensure effective resource utilization. Strengthening incentive mechanisms, such as financial rewards and policy support for faculty participation in technology transfer activities, is also critical.

3 **Differentiated strategies by institutional type**

Given the significant performance differences among university types, tailored strategies are necessary:

- Normal and Medical & Pharmaceutical Universities: Policies should aim to consolidate existing strengths and promote closer integration with local economies and industries to enhance the practical application of research outcomes.
- Comprehensive and Science & Engineering Universities: Efforts should focus on improving TTA efficiency by fostering interdisciplinary collaboration and deepening engagement with industry partners, increasing the rate of successful technology transfers.
- Agricultural and Forestry Universities: These universities should prioritize the development and dissemination of advanced agricultural technologies. Emphasis should be placed on bridging technological gaps and accelerating the adoption of innovative solutions in the agricultural sector.

## Supporting information

S1 File Overall, stage, and period-stage efficiency of 67 research universities. (7Z)

S1 Appendix All abbreviations and nomenclature used in this study are summarized below. (DOCX)
