# Supplementary material for: Efficiency analysis of 67 Chinese research universities considering inter-university heterogeneity: Evidence from a meta-frontier network SBM DEA model
Source: PLoS One. 2025 Sep 18;20(9):e0331923. doi: 10.1371/journal.pone.0331923 (PMC12445460; doi:10.1371/journal.pone.0331923)
Supplement: S1 Appendix — (DOCX) [file pone.0331923.s002.docx]

Appendix A. All abbreviations and nomenclature used in this study are summarized below

Table A1. Abbreviations and full name

| Abbreviation | Full name |
| --- | --- |
| DEA | Data Envelopment Analysis |
| DMUs | Decision-Making Units |
| CCR | Charnes, Cooper, and Rhodes |
| BCC | Banker, Charnes, and Cooper |
| VRS | Variable Returns to Scale |
| CRS | Constant Returns to Scale |
| R&D | Research and Development |
| TTA | Technology Transfer and Application |
| TGR | Technology Gap Ratio |
| MI | Management Inefficiency |
| TGRI | technological gap inefficiency |
| MF | Meta-Frontier |
| GF | Group-Frontier |
| MFE | Meta-Frontier Efficiency |
| GFE | Group-Frontier Efficiency |
| GMI | Group Managerial Inefficiency |
| SBM | Slack-based Measure |
